# Behavioral Characteristics of China’s NEET-Prone University Students and Graduates: A Survey from Southwest China

**DOI:** 10.3390/bs14020098

**Published:** 2024-01-28

**Authors:** Lu Zhao, Yang Li, Ao Yu, Weike Zhang

**Affiliations:** 1 School of Marxism, Sichuan University, Chengdu 610065, China; zhaolu_sl@scu.edu.cn; 2 Youth League Committee, Sichuan University, Chengdu 610065, China; li-yang@scu.edu.cn; 3 School of Economics, Sichuan University, Chengdu 610065, China; 4 School of Public Administration, Sichuan University, Chengdu 610065, China; zhangwk@scu.edu.cn

**Keywords:** NEET, youth, Chinese student, NEET-prone student, further education

## Abstract

The NEET phenomenon (not in education, employment or training) has significant implications, both for individuals and society at large. While China’s higher education students are particularly susceptible to becoming NEET, relatively little attention has been given to understanding this issue. To address this research gap and contribute to the study of NEET in China, this paper collected a total of 12,616 samples from current higher education students and those who graduated within the past three years from universities in seven provinces of Southwest China, finding that 21.91% of the students surveyed fall into the NEET-prone student category. The underlying factors contributing to NEET-prone status are identified through logit regression analysis and categorized into three levels: individual, family, and society. At the individual level, factors such as personal ability, confidence in job-hunting, and attitude towards NEET significantly influence a student’s likelihood of being NEET-prone. Family-level factors include being an only child, consumption level, economic dependence on family members, and the presence of NEET relatives. And social-level factors encompass school provision (or non-provision) of employment services, the number of employment services offered, and the possibility of obtaining loans from society. Finally, this paper concludes by offering recommendations, which are drawn from individual, family, and social perspectives, to help Chinese higher education students avoid NEET status.

## 1. Introduction

Youths who are not in education, employment or training are commonly referred to as NEETs [1]. The concept of NEET originated in the United Kingdom and then spread rapidly to developed countries such as Japan and the United States. In recent years, NEETs have exhibited new characteristics [2]. Some youths do not unwillingly become NEETs, and instead voluntarily become part of this category in what is known as the post-materialist era, when young people focus more on their inner spiritual needs and self-expression, and simultaneously diminish their enthusiasm for work and suppress their material desires [3,4,5]. On one hand, some youths may appear to have jobs but are essentially marginalized because their nominal employment is merely a passive response to external demands; on the other hand, some youths postpone entering the labor force by pretending to prepare for further education or employment. In either negatively delaying employment or actively choosing to be under-employed, they are nonetheless gradually classified as NEET [6].

Recent research indicates that the global average NEET rate in 2020 is 15.3% for males and 31.1% for females. The pandemic has further increased this rate, with global youth employment falling by 8.7% in 2021 [7]. Being NEET does not only signify unemployment for young people but also implies a loss of purpose in life [8]. Engaging in activities such as having a job or pursuing education helps young individuals to establish connections with society, positioning themselves in a way that prevents marginalization [9,10,11]. Moreover, the societal costs of the phenomenon extend beyond individual suffering, and include the loss of a potential workforce and the erosion of economic dynamism [12]. It also increases the risk of instability, including drug use and suicide [13].

**Figure 1:**
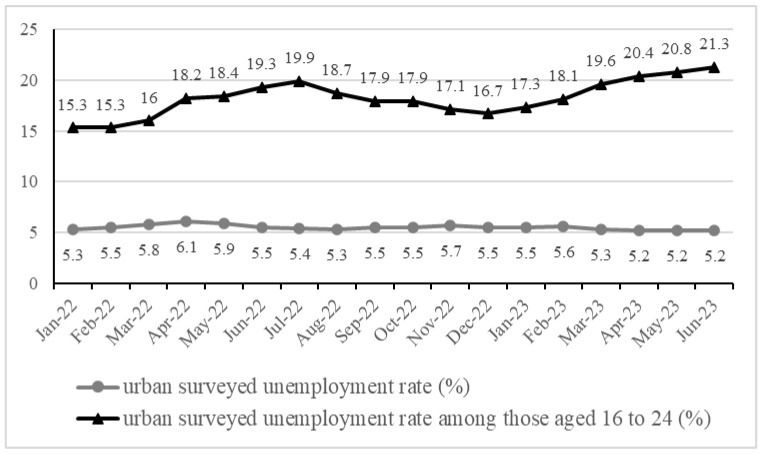
China’s recent urban surveyed unemployment rate. Source: National Bureau of Statistics, https://data.stats.gov.cn/easyquery.htm?cn=A01 (accessed on 20 October 2023).

Although significant progress has been made in researching the NEET phenomenon in developed countries, little attention has been given to China [14]. NEET in China is closely related to the high youth unemployment rate, which not only compels Chinese youths to become NEET but also undermines their confidence and thereby influences their active decision to embrace NEET status. Figure 1 illustrates China’s recent urban surveyed unemployment rate, showing that while the unemployment rate of all age groups is stable at 5 to 6 percent, it rises to around 20 percent for youths aged 16–24. The two primary factors that contribute to the challenges faced by youths hoping to secure employment are the stagnation of the services sector after the pandemic (the primary source of youth employment), and the diploma inflation that has resulted from the Chinese enrollment expansion reform.

Students in higher education, a significant demographic of Chinese youth, are particularly susceptible to becoming NEET. Firstly, Chinese higher education students often find themselves unprepared and are uncertain about their future prospects when they realize that exams and grades do not solve all their problems. Throughout their childhood, and particularly in high school, they were told that if they survived the gaokao (the national college entrance exam), all their problems would automatically disappear. 

Secondly, the substantial disparity between their expectations and reality is another crucial factor that leads them to become NEET. They have been consistently exposed to misleading messages from self-media, which falsely suggest that because it is easy to secure high-paying jobs, low-paying jobs are not worth pursuing. Thirdly, excessive parental indulgence also contributes significantly to the prevalence of NEET among Chinese students. Parents overindulging their children, a Chinese cultural practice, can mean they lack the awareness and skills necessary for employment—among other things, this underlines that the NEET phenomenon among Chinese higher education students is not only rooted in common causes shared with developed countries, but also in China’s unique cultural background. It is of the utmost importance to recognize this when investigating Chinese students, especially those prone to becoming NEET.

This study hand collects a total of 12,616 samples from current higher education students and those who graduated within the past three years from universities in seven provinces of Southwest China. The aim of this study is to investigate the scale and characteristics of the phenomenon, and to identify its underlying reasons in Chinese students who are prone to becoming NEET. Several conclusions are drawn by this research. Firstly, 21.91% of the students fall into the NEET-prone category; there are a higher proportion of males, compared to females; a larger number of 19 or 20-year-olds, compared to other age groups; a greater representation of rural students, compared to urban students; and a higher prevalence among students with limited educational backgrounds. 

This study uses the Logit regression method to empirically demonstrate that the phenomenon of Chinese NEET-prone higher education students is influenced by a combination of individual, family, and societal factors:: individual-level factors include personal ability, confidence in job-hunting, and attitude towards NEET; family-level factors include whether the student is an only child, consumption level, economic dependence on family members, and if any relatives are NEET. And social-level factors include whether schools provide employment services; the number of employment services provided by schools; and whether the student has any loans from society.

This paper makes several contributions to the existing literature. Firstly, it expands previous research of NEET by specifically focusing on Chinese higher education students who are at risk of becoming NEET. This targeted approach is of utmost importance for several reasons: (1) according to China’s National Bureau of Statistics (NBS), the number of higher education students in China reached 58 million in 2022. Given the substantial size of this population, it is essential to examine this specific subgroup. (2) higher education students are at a critical stage in their personal development, and may experience confusion about their future paths, which could potentially lead them into the NEET category. (3) in addition to the common factors contributing to worldwide NEET, there are also unique cultural aspects in China that contribute to this phenomenon. For instance, families in China play a more significant role in encouraging young people to become NEET, compared to other countries. Secondly, this paper places significant emphasis on collecting a substantial sample, which greatly enhances the study of NEET-prone Chinese students and contributes to the existing research of NEET. The use of hand collected survey data makes it possible to identify NEET-prone students, particularly those who lack the willingness to seek employment, by directly inquiring about their life plans by using questionnaires.

The paper is structured as follows: Section 2 provides a comprehensive literature review; Section 3 discusses the methodology, including the method, sample, variable and model; Section 4 presents the results and discusses factors that contribute to Chinese students becoming NEET; and the concluding section offers conclusions and corresponding suggestions.

## 2. Literature Review

NEET research originated in the United Kingdom and quickly spread to developed countries such as France, Germany, Spain, and the United States. In Europe, the NEET rate for individuals aged 15 to 29 was 14% in 2021, with Italy having the highest rate (23.1%) [15]. The United States has also experienced a comparable situation, with a NEET rate of 11.7% among individuals aged 16 to 24 in 2016. However, in the second quarter of 2020, following on from the COVID-19 pandemic, this was estimated to have increased to between 20% and 28% [16].

Being NEET presents numerous foreseeable risks. At the national level, the NEET phenomenon contributes to an increased likelihood of poverty and inequality, as it results in a decline in social engagement and participation [17]; on an individual level, NEET are more prone to feelings of inferiority and isolation [18], which often lead to decreased emotional well-being and, in severe cases, to the development of psychotic disorders and self-directed violence that is difficult to control [19], particularly when parents, relatives, and friends are critical [20]. In addition, Ralston et al. [21] support the concept of “employment scarring” among NEET, suggesting that individuals who were previously NEET continue to show low work-related motivation and poor job performance for an extended period of time.

The causes of NEET status in developed countries have been extensively discussed by academic researchers. At the macro level, NEET status is primarily attributed to insufficient demand in the labor market [22]. The NEET population tends to fluctuate with youth unemployment rates, with significant increases observed in economic recessions. Young individuals who lack employability skills are particularly vulnerable to becoming NEET. [23]. The COVID-19 pandemic has further exacerbated the issue, as deteriorating labor market conditions have led to an increase of NEET [24]. Another crucial factor contributing to NEET status is the availability of educational support and vocational training, which aims to enhance individuals’ work ability and job-hunting skills. For instance, Lorinc et al. [25] argue that the fundamental cause of NEET lies in the quantity and quality of education and training provided by society, and suggest that individual difficulties in education and training reflect the lack of these social services. Additionally, social factors, such as equality, play a significant role in NEET rates, as emphasized by Lallukka et al. [26], highlighting the importance of social equity in improving employment prospects for young people.

At the micro level, research indicates that NEET is associated with adverse family situations [27], early school dropout, low literacy respectively [28,29], poor health status [30], and addiction-related behaviors [31]. For instance, Caroleo et al. [10] find that economically disadvantaged European youth are more susceptible to becoming NEET, while Berlin et al. [32] highlight poor school performance, including early school leaving, frequent exam failures, and low class attendance, as primary causes of NEET in Denmark, Finland, and Sweden. Furthermore, Ringbom et al. [30] demonstrate the impact of various psychiatric and neurodevelopmental disorders on NEET, and observe psychosis and autism spectrum disorder exert the greatest influence. Rodwell et al. [33] present evidence of an increased likelihood of NEET status among marijuana addicts by drawing on a cohort study of 1938 participants from Victoria, Australia. And individual characteristics, such as age, gender, and intelligence quotient [34,35] can also contribute to NEET status.

The NEET phenomenon also exists in developing countries and emerging markets [36,37,38], although research of NEET in these contexts is relatively limited. China, which has a significant population of NEETs, has received limited attention from academia. To et al. [39] conduct interviews with 14 parents of NEETs in Hong Kong and reveal that parents’ negative personal experiences can lead to irrational parenting behaviors, such as blaming their children for their failures and imposing their own ideas on them, which can increase the likelihood of their children becoming NEET. Li et al. [40] conduct semi-structured interviews with 30 NEETs in Hong Kong, China, and identify three psychological pathways out of NEET status (finding a balance between ideals and reality, seeking solace and support from others about insoluble predicaments, and regaining life motivation). And Yang [14] uses logistic regression to analyze the impact of personal traits, including gender, marital status, and education, on the likelihood of becoming a NEET, concluding that females, particularly those who are married, face a higher risk of becoming NEET, and that education is a protective factor against NEET status.

In conclusion, while the NEET phenomenon in developed countries has been extensively researched, a lack of studies have specifically focused on emerging economies such as China. It is important to acknowledge that NEETs in China have distinct characteristics, compared to counterparts in developed countries. For instance, they rely heavily on their families for support, which is one reason why it is problematic to generalize conclusions drawn from developed country studies to the Chinese context.

Finally, despite the role of higher education being crucial in shaping an individual’s life perspective, the existing literature gives insufficient attention to the NEET phenomenon among higher education students in the existing literature. Accordingly, this paper aims to address this research gap by examining the NEET phenomenon among higher education students in China, with particular emphasis on NEET-prone students.

## 3. Methodology

### 3.1. Method

The research methods utilized in the field of social sciences can be broadly classified into qualitative and quantitative approaches. In this study, we use quantitative research methods to examine the behavioral characteristics of Chinese students who are prone to NEET status. This use of quantitative research methods allows an objective and precise investigation, reducing potential biases and subjective perceptions, and also effectively identifies the multifaceted factors, which operate at the individual, familial, and societal levels, that influence the transition of Chinese students into NEET status,

### 3.2. Sample

This study aims to investigate the characteristics and underlying factors of NEET-prone students in China by collecting samples from current higher education students and students who, in the past three years, graduated from universities in seven provinces of Southwest China (Guangxi, Gansu, Guizhou, Qinghai, Xinjiang, Yunnan, and Sichuan). In engaging with the period from March to July 2023, this paper employs a combination of stratified sampling and random sampling methods to conduct a questionnaire survey, of both current students and recent graduates, across 55 colleges and universities within the aforementioned provinces. Specifically, this study randomly selects a proportional representation of universities or colleges, which is based on the number of universities in each province, and includes double-first-class universities, ordinary undergraduate universities, and junior colleges (or higher vocational colleges). Random sampling is then subsequently used to distribute questionnaires to current students and recent graduates, across different types of universities and colleges. This study distributes a total of 13,500 questionnaires and returns a total of 12,776 questionnaires, before extracting 12,616 valid questionnaires, an effective recovery rate of 93.45%.

In order to ensure the questionnaire’s reliability and validity, this study initially applied Statistical Product and Service Solution (SPSS22.0) to conduct a reliability analysis of observable variables. The Cronbach values for all variables ranged between 0.80 and 0.90, surpassing the minimum criterion of 0.70, indicating that the study questionnaire has high credibility and meets the requirements of the reliability test. The construct validity of the questionnaire was then assessed by using the factor analysis function in the SPSS software’s factor analysis module, producing results that demonstrate that all of the criteria of the construct validity test (including the eigenvalue of the common factor exceeding 1; the cumulative variance of the common factor surpassing 70%, the standard factor loading of each question being greater than 0.5, and the AVE value being higher than 0.5 were met. These criteria (including the eigenvalue of the common factor exceeding 1, the cumulative variance of the common factor surpassing 70%; the standard factor loading of each question being greater than 0.5; and the AVE value being higher than 0.5). Due to space limitations, the specific test process and its results are not listed.

### 3.3. Method

#### 3.3.1. Variable

The dependent variable in this study is NEET-prone students, expressed as NEET-prone, which is defined as students who answer “temporarily fail to plan for further education, employment, or training” when asked about the next step in their future plans. This variable takes a value of 1 if the student meets this criterion, and is otherwise 0.

The independent variables consist of individual-level, family-level, and social-level variables. The individual level examines personal ability (Ability), confidence in job-hunting (Confidence), and attitude toward NEET (Attitude). Specifically, Ability is measured through self-evaluation of seven aspects, including expertise, expression, teamwork, innovation, stress tolerance, autonomous learning, and organization management, which are each scored on a scale of 1 to 5. Confidence is assessed by asking students to rate how easy they believe it is to achieve their employment goals, by indicating response options ranging from 1 to 5 (representing very difficult, not easy, general, relatively easy, and very easy, respectively). Attitude is determined by how long a student can accept being NEET, and is categorized into six levels: (within half a year, half to one year, one to two years, two to three years, three years or more, and whatever).

The family-level variables include four factors: whether the student is an only child (Only-child), consumption level (Consumption), economic dependence on family members (Dependence), and whether any relatives are NEET (Relative-NEET). To be specific, Only-child takes a value of 1 if the student is an only child, and is otherwise 0. Consumption is determined based on monthly expenses, with six categories (1000 RMB, 1001 to 1500 RMB, 1501 to 2000 RMB, 2001 to 2500 RMB, 2501 to 3000 RMB, and above 3000 RMB, respectively). Dependence is coded as 1 if the student’s living expenses are mainly provided by family members, and is otherwise 0. Relative-NEET is assigned a value of 1 if the student has any relatives who are NEET.

Three social-level variables are considered: whether schools provide employment services (Service), the number of employment services provided by schools (Num-service), and whether the student have any loans from society (Loan). Specifically, Service is a binary indicator that determines whether the school offers any of six employment services: career planning courses, extracurricular activities related to career planning (e.g., lectures, consultations, training, tutoring), release recruitment information, organize recruitment fairs, internship opportunities, and provide employment channels or guidance for graduates. And Num-service quantifies the count of employment services provided by the school. Loan is assigned a value of 1 if the student has obtained loans from banks, lending platforms (e.g., Alipay, WeChat, P2P), relatives, or friends, and is 0.

The control variables include gender (Gender), age (Age), nationality (Nationality), marriage (Marriage), household registration (Registration), and family income level (Family-income). Gender is set to 0 for females and 1 for males. Age is the student’s age; Nationality is 0 for Han students and 1 for all others. Marriage is 0 for unmarried students and 1 for those who are married; Registration is 0 for rural students and 1 for urban counterparts; Family-income has a value of 1 to 5, which indicate the annual family income of rural students below 12,000 RMB, 12,001 to 15,000 RMB, 15,001 to 20,000 RMB, 20,001 to 30,000 RMB, and above 30,000 RMB respectively; and urban students whose annual family income is below 24,000 RMB, 24,001 to 36,000 RMB, 36,001 to 45,000 RMB, 45,001 to 60,000 RMB, and above 60,000 RMB, respectively.

The descriptive statistics of the above variables are shown in Table 1.

#### 3.3.2. Model

This study uses the logit regression model to examine the factors that contribute to Chinese students’ propensity to become NEET-prone individuals. The study’s dependent variable is binary, as demonstrated in Model (1):

(1) $$\ln(\frac{P(NEET-prone_{i})}{1-P(NEET-prone_{i})})=\beta_{0}+\beta_{1}x_{i}+\lambda C$$

where *P*(*NEET*-*prone_i_*) represents the probability of student *i* becoming a NEET-prone individual; *x* represents the independent variable (with *β*_1_ as is its coefficient). *x* includes Ability, Confidence, Attitude, Only-child, Consumption, Dependence, Relative-NEET, Service, Num-service, and Loan, and these variables are added separately to Model (1); and *C* is the control variables, including Gender, Age, Nationality, Marriage, Registration, and Family-income.

## 4. Result and Discussion

### 4.1. Distribution of Chinese NEET-Prone Students

Of the 12,616 samples, a total of 2764 students (21.91%), are categorized as NEET-prone, a proportion comparable to developed countries, as reported by [15]. Table 2 displays the distribution of these samples and NEET-prone students across six variables: gender, age, nationality, household registration, educational background, and school level.

For gender, it is surprising to see that,, despite only 39.47% of the samples being male, 56.91% of NEET-prone students are male, which indicates that Chinese male students are more likely to be NEET-prone than their female counterparts. This contradicts the research of [14], who suggests that Chinese women are more vulnerable to NEET, a discrepancy that could be attributed to differences in the samples used: this study specifically focuses solely on Chinese higher education students, whereas Yang [14] examines all youth groups. The higher likelihood of male students becoming NEET-prone may also stem from them often shouldering greater responsibilities in supporting their families, leading them to teeter between employment and further education, their natural advantage in job prospects notwithstanding.

For age, Chinese students who are 19 or 20 years-old are more susceptible to becoming NEET-prone individuals, as they are still in the early stages of life planning. For nationality, Han students are more prone to slipping into NEET status compared to other ethnic minority groups. For household registration, rural students make up 72.47% of the total, with rural NEET-prone students accounting for 85.46%, which indicates that rural students in China are more likely to become NEET-prone. These findings are not surprising, given that rural students receive significantly less support from their families, compared to their urban counterparts. And finally, students with educational disadvantages and those who attend lower-level schools are more likely to fall into NEET status.

### 4.2. Key Factors in Chinese Students Falling into NEET

#### 4.2.1. Results of the Individual-Level Factors

Table 3 presents the regression results for individual-level factors, namely personal ability (Ability), confidence in job-hunting (Confidence), and attitude toward NEET (Attitude). The results of Ability, Confidence, and Attitude are shown in Columns (1) and (2), Columns (3) and (4), and Columns (5) and (6), respectively; Columns (1), (3) and (5) are results without control variables, and Columns (2), (4), and (6) are those with.

As shown in Columns (1) and (2), the coefficients of Ability are significantly negative at the 1% level, which indicates that students with higher personal ability are less likely to become NEET-prone students. Personal ability gives students more further education and employment opportunities, which in turn motivates them to plan for the future, meaning they avoid falling into NEET.

The coefficients of Confidence in Columns (3) and (4) are smaller than 0 at the 1% significance level, which shows confidence in job-hunting contributes to the avoidance of NEET. Confidence is extremely critical to students facing an uncertain future, as it gives them the courage to put effort in and struggle, benefitting them in both education and employment.

The coefficients of Attitude in Columns (5) and (6) are negative at the 1% significant level, which evidences that students who are negatively predisposed to NEET face less risk of falling into this category. Students’ attitudes toward NEET define their choices, and their negative predisposition to NEET empowers them to tap into their potential and achieve competence in both further education and employment.

The results of the control variables are consistent with those in Section 3.2. The significant coefficients of Gender reiterate that male students are more likely to become NEET-prone students than their female peers; the coefficients of Age are negative at the 1% significant level, showing older students are less likely to become NEET-prone students, which is due to them being older and better able to cope with stress, which ensures their survival; Nationality shows significant negative coefficients, echoing the observation that Han students are more likely to be NEET-prone students than others; Marriage shows negative coefficients that are insignificant, which may be because there are too few married samples; and both Registration and Family-income are significantly negative, sufficiently demonstrating that urban students and students with superior family conditions are less likely to be NEET-prone students.

#### 4.2.2. Results of the Family-Level Factors

Table 4 reports the regression results of the family-level factors including, only child status (Only-child), consumption level (Consumption), economic dependence on family members (Dependence), and whether any relatives are NEET (Relative-NEET).

The coefficients of Only-child in Columns (1) and (2) show significant negative signs at the 5% level, meaning that if the student is the only child in their family, they are less likely to be a NEET-prone student. On the one hand, the only children can receive more financial and energetic support from their families, and this material support and guidance will considerably aid their pursuit of employment and further education. On the other hand, only children shoulder higher family expectations, pushing them to work harder and reducing the likelihood they will become NEET-prone students.

The Consumption in Columns (3) and (4) has negative coefficients at the 1% significant level, suggesting that students with a higher consumption level are less likely to be NEET-prone than lower consumption-level peers. The underlying reason may be because higher consumption levels can ensure more extracurricular training and provide more learning materials, enabling them to steer clear of NEET.

The coefficients of Dependence in Columns (5) and (6) are significantly positive at the 1% level, indicating that when students are more dependent on their family members, they are more likely to become NEET-prone. Overdependence on their family members causes them to lose the courage to make their own choices and indulge in the pleasure of self-creation, which both make them more likely to become NEET.

The coefficients of Relative-NEET in Columns (7) and (8) are significantly negative at the 1% level, showing that students are less likely to be NEET if they have a NEET relative. At first glance, it may be assumed that NEET relatives set a bad example, increasing the likelihood of students becoming NEET-prone. But in actual fact, comparisons between Chinese parents, and especially with their in-law relatives, results in NEET relatives instead being used as negative examples, reducing the likelihood children will become NEET.

In referring to the control variables, we see there is no obvious difference with those in Section 4.2.1 in the sign and significance of coefficients, supporting the claim the preceding conclusions are robust.

#### 4.2.3. Results of the Social-Level Factors

Table 5 reports the regression results of the social-level factors, including school provision of employment services (Service), number of employment services provided by schools (Num-service), and the obtaining of loans from society (Loan).

The coefficients of Service in Columns (1) and (2) are negative at the 1% significance level, and those of Num-service in Columns (3) and (4) are significantly negative. These results provide sufficient evidence that school employment service provision can help students escape NEET, by helping students to identify their strengths and weaknesses, providing training in job-hunting skills, and compensate for a lack of job information, which all reduce the possibility of becoming NEET.

As shown in Columns (5) and (6), the coefficients of Loan are significantly negative at the 1% level, meaning students who receive loans are less likely to become NEET. On the one hand, receiving loans, and especially student loans, helps students complete their education without wasting a lot of time on low value work, reducing the likelihood of NEET. On the other hand, the pressure to pay back their loans forces them to struggle for both employment and further education. In referring to the control variables, we see similar results to those previously obtained, which confirms the preceding conclusions again.

When examining the factors that influence the NEET status of Chinese students, we individually included each variable in the regression model. However, this raises concerns about potential omitted variables, as all factors may have a simultaneous impact on the dependent variable. To address this, we integrated all factors across various levels into the model and conducted regression analysis again. The outcomes of these regressions are presented in Table 6. In Column (1), we incorporated all the individual-level factors. In Column (2), we introduced all the family-level factors. In Column (3), we considered all the social-level factors. And in Column (4), we simultaneously included these factors at all levels. The regression results for all factors consistently show statistically significant findings, which both echoes and reinforces what has previously been observed. 

## 5. Conclusions and Suggestions

This study aims to investigate the phenomenon of Chinese NEET-prone students by using a sample of 12,616 students who are both currently enrolled in higher education and also graduated within the past three years. These samples are collected by hand from seven provinces in Southwest China. The findings reveal that 21.91% of students are classified as NEET-prone, a result influenced by individual, family, and societal factors. At the individual level, factors such as personal ability, confidence in job-hunting, and attitude towards NEET have an impact on proneness to NEET status, and students with higher personal ability, stronger confidence, and a negative attitude to NEET are less likely to be categorized as NEET-prone. At the family level, factors such as being an only child, consumption level, economic dependence on family members, and the presence of NEET relatives also influence the likelihood of NEET proneness. Students who are only children, have a higher consumption level, rely more on family members for financial support, and have NEET relatives are less likely to be NEET-prone. And at the social level, factors such as the school provision of employment services, the number of school employment services offered, and loans from society also play a role. Students whose schools provide employment services and a greater number of services, and who receive loans from society are less likely to be NEET-prone.

In drawing on these findings, this paper proposes several recommendations that will help Chinese students to overcome NEET. From an individual perspective, it is crucial to prioritize the development of students’ comprehensive abilities as a way of combatting NEET. Higher education students should break free from the exam-oriented thinking ingrained in their secondary education and focus on cultivating skills in various areas, including not just knowledge retention but also information retrieval, teamwork, communication, and writing. Additionally, instilling the right values in students is of the utmost importance in addressing NEET. For instance, fostering independence in decision-making, building confidence in the face of failure, and nurturing a sense of resilience are all essential qualities for Chinese students.

From a familial standpoint, three types of efforts can be made to combat NEET. Firstly, parents should provide their children with increased financial and emotional support. Financial assistance enables students to afford job training and learning resources, while emotional support helps them gain a better understanding of their strengths and weaknesses, enabling them to identify suitable career paths at an earlier stage. 

Secondly, parents should involve their children in productive activities, such as work-study programs, on the condition that this does not impede their studies. Early exposure to such activities benefits students by helping them find meaning and enjoyment in work and preparing them for future employment. Finally, fostering a healthy competitive environment among family members can be beneficial. In China, family members are often compared to each another, and a positive competitive mechanism can help individuals to better understand themselves and avoid the path to NEET.

From a social perspective, schools should optimize their curriculum system and enhance career guidance. In addition to increasing the dissemination of recruitment information, schools should prioritize curriculum reform centered on employment and further education. This approach will foster students’ awareness and enhance their employability and further education prospects. The government should also simultaneously provide increased support (including financial assistance such as student loans, technical assistance that reduce information asymmetry between students and enterprises, and policy measures that safeguard employment rights), as this will help students avoid becoming NEET. 

## Figures and Tables

**Table 1:** Descriptive statistics.

Type	Variable	N	Mean	S.D.	Min	Max
Dependent variable	NEET-prone	12,616	0.219	0.414	0	1
Independent variable	Ability	12,616	24.950	6.007	7	35
	Confidence	12,616	2.704	0.970	1	5
	Attitude	12,616	4.708	1.843	1	6
	Only-child	12,616	0.265	0.441	0	1
	Consumption	12,616	2.143	1.080	1	6
	Dependence	12,616	0.627	0.484	0	1
	Relative-NEET	12,616	0.215	0.411	0	1
	Service	12,616	0.935	0.246	0	1
	Num-service	12,616	3.049	1.911	0	6
	Loan	12,616	0.226	0.418	0	1
Control variable	Gender	12,616	0.395	0.489	0	1
	Age	12,616	19.720	1.534	15	45
	Nationality	12,616	0.181	0.385	0	1
	Marriage	12,616	0.007	0.083	0	1
	Registration	12,616	0.275	0.447	0	1
	Family-income	12,616	2.300	1.518	1	5

**Table 2:** Distribution results.

Type (1)	Samples N (%) (2)	NEET-Prone Students N (%) (3)
(1) Gender		
Male	4980 (39.47%)	1573 (56.91%)
Female	7636 (60.53%)	1191 (43.09%)
(2) Age		
18 and below	2005 (15.89%)	415 (15.01%)
19	4221 (33.46%)	1025 (37.08%)
20	3791 (30.05%)	875 (31.66%)
21	1564 (12.40%)	318 (11.51%)
22 and above	1035 (8.20%)	131 (4.74%)
(3) Nationality		
The Han nationality	10,331 (81.89%)	2362 (85.46%)
Others	2285 (18.11%)	402 (14.54%)
(4) Household registration		
Rural	9143 (72.47%)	2274 (82.27%)
Urban	3473 (27.53%)	490 (17.73%)
(5) Educational background		
Associate degree	9558 (75.76%)	2450 (88.64%)
Bachelor degree	2793 (22.14%)	301 (10.89%)
Master’s degree	208 (1.65%)	11 (0.40%)
Doctor’s degree	57 (0.45%)	2 (0.07%)
(6) Level of school		
College or vocational college	9252 (73.34%)	2393 (86.58%)
General undergraduate universities	497 (3.94%)	80 (2.89%)
Double first-class universities	2867 (22.73%)	293 (10.60%)

The % in Column (2) refers to the proportion of the various types of samples in the total of 12,616 samples; the % in Column (3) represents the proportion of the various types of NEET-prone students in the total of 2764 NEET-prone students.

**Table 3:** Regression results of the individual-level factors.

	Personal Ability		Confidence in Job Hunting		Attitude towards NEET	
	(1)	(2)	(3)	(4)	(5)	(6)
Ability	−0.050 ***	−0.048 ***				
	(0.004)	(0.004)				
Confidence			−0.229 ***	−0.221 ***		
			(0.023)	(0.023)		
Attitude					−0.107 ***	−0.101 ***
					(0.011)	(0.011)
Gender		0.255 ***		0.254 ***		0.168 ***
		(0.045)		(0.045)		(0.045)
Age		−0.090 ***		−0.098 ***		−0.091 ***
		(0.017)		(0.017)		(0.017)
Nationality		−0.392 ***		−0.394 ***		−0.380 ***
		(0.062)		(0.062)		(0.062)
Marriage		−0.509		−0.475		−0.664 *
		(0.362)		(0.362)		(0.361)
Registration		−0.645 ***		−0.644 ***		−0.680 ***
		(0.057)		(0.057)		(0.056)
Family-income		−0.077 ***		−0.077 ***		−0.076 ***
		(0.016)		(0.016)		(0.016)
_cons	−0.051	1.973 ***	−0.667 ***	1.533 ***	−0.777 ***	1.312 ***
	(0.089)	(0.341)	(0.063)	(0.340)	(0.054)	(0.333)
N	12,616	12,616	12,616	12,616	12,616	12,616
Log likelihood	−6534.94	−6385.24	−6581.60	−6428.30	−6587.34	−6434.42
LR chi2	195.85	495.24	102.53	409.12	91.05	396.89
Prob > chi2	0.000	0.000	0.000	0.000	0.000	0.000
Pearson chi2	130.48	3045.30	77.76	1374.33	25.01	1381.07
Prob > chi2	0.000	0.295	0.000	0.001	0.000	0.011
Pseudo R^2^	0.015	0.037	0.008	0.031	0.007	0.030

Standard errors are in parentheses; *** *p* < 0.01, * *p* < 0.1; LR chi2 demonstrates the joint significance of both independent and control variables, and a *p*-value below 0.1 indicates a high level of joint significance. As shown, all models are jointly significant; Pearson chi2 demonstrates the goodness of fit of the model, and when its *p*-value exceeds 0.1, it indicates a satisfactory fit of the model; As shown, the majority of models do not exhibit an ideal goodness of fit, which could be attributed to the insufficient inclusion of influencing factors in the model.

**Table 4:** Regression results of the family-level factors.

	Only Child?		Consumption Level		Economic Dependence on Family Members		Any NEET Relatives ?	
	(1)	(2)	(3)	(4)	(5)	(6)	(7)	(8)
Only-child	−0.326 ***	−0.135 **						
	(0.051)	(0.056)						
Consumption			−0.159 ***	−0.076 ***				
			(0.022)	(0.023)				
Dependence					0.349 ***	0.347 ***		
					(0.007)	(0.007)		
Relative-NEET							−0.261 ***	−0.234 ***
							(0.055)	(0.056)
Gender		0.223 ***		0.204 ***		0.046 ***		0.211 ***
		(0.044)		(0.044)		(0.007)		(0.044)
Age		−0.091 ***		−0.091 ***		−0.002		−0.090 ***
		(0.017)		(0.017)		(0.002)		(0.017)
Nationality		−0.385 ***		−0.348 ***		−0.069 ***		−0.371 ***
		(0.062)		(0.062)		(0.009)		(0.062)
Marriage		−0.544		−0.540		0.044		−0.532
		(0.360)		(0.359)		(0.041)		(0.360)
Registration		−0.633 ***		−0.686 ***		−0.082 ***		−0.670 ***
		(0.059)		(0.058)		(0.008)		(0.056)
Family-income		−0.082 ***				−0.015 ***		−0.085 ***
		(0.016)				(0.002)		(0.016)
_cons	−1.190 ***	0.861 ***	−0.938 ***	0.808 **	0.000	0.088 *	−1.218 ***	0.881 ***
	(0.025)	(0.330)	(0.049)	(0.330)	(0.006)	(0.045)	(0.024)	(0.330)
N	12,616	12,616	12,616	12,616	12,616	12,616	12,616	12,616
Log likelihood	−6600.21	−6447.13	−6569.20	−6423.67			−6612.60	−6448.62
LR chi2	65.29	371.46	127.32	418.39			40.52	368.48
Prob > chi2	0.000	0.000	0.000	0.000			0.000	0.000
Pearson chi2	0.00	671.32	23.83	1638.49			0.00	736.95
Prob > chi2	0.000	0.030	0.000	0.130			0.000	0.100
Pseudo R^2^	0.003	0.024	0.004	0.023			0.002	0.025
Adj R^2^					0.167	0.185		

Standard errors are in parentheses; *** *p* < 0.01, ** *p* < 0.05, * *p* < 0.1; Family-level is not introduced when analyzing the impact of consumption level, as it is highly correlated with Consumption; The result of economic dependence on family members is based on the OLS regression rather than the Logit regression because Dependence is automatically culled when logit regression is used; LR chi2 demonstrates the joint significance of both independent and control variables, with a *p*-value below 0.1 indicating a high level of joint significance—as shown, all models are found to be jointly significant; Pearson chi2 demonstrates the goodness of fit of the model, with a *p*-value above 0.1 indicating a satisfactory fit of the model—as shown, the majority of models do not exhibit an ideal goodness of fit, which can perhaps be attributed to influencing factors being insufficiently included in the model.

**Table 5:** Regression results of the social-level factors.

	Do Schools Provide Employment Services?		Number of Employment Services Provided by Schools		Any Loans from Society?	
	(1)	(2)	(3)	(4)	(5)	(6)
Service	−0.647 ***	−0.591 ***				
	(0.077)	(0.079)				
Num-service			−0.130 ***	−0.117 ***		
			(0.012)	(0.012)		
Loan					−0.340 ***	−0.397 ***
					(0.055)	(0.057)
Gender		0.196 ***		0.198 ***		0.216 ***
		(0.044)		(0.044)		(0.044)
Age		−0.093 ***		−0.095 ***		−0.086 ***
		(0.017)		(0.017)		(0.017)
Nationality		−0.369 ***		−0.381 ***		−0.279 ***
		(0.062)		(0.062)		(0.063)
Marriage		−0.587		−0.604 *		−0.478
		(0.360)		(0.361)		(0.360)
Registration		−0.670 ***		−0.651 ***		−0.707 ***
		(0.056)		(0.056)		(0.056)
Family-income		−0.079 ***		−0.072 ***		−0.096 ***
		(0.016)		(0.016)		(0.016)
_cons	−0.673 ***	1.423 ***	−0.893 ***	1.239 ***	−1.200 ***	0.850 **
	(0.074)	(0.339)	(0.039)	(0.335)	(0.024)	(0.330)
N	12,616	12,616	12,616	12,616	12,616	12,616
Log likelihood	−6600.21	−6447.13	−6569.20	−6423.67	−6612.60	−6448.61
LR chi2	65.29	371.46	127.32	418.39	40.52	368.48
Prob > chi2	0.000	0.000	0.000	0.000	0.000	0.000
Pearson chi2	0.00	671.32	23.83	1638.49	0.00	736.95
Prob > chi2	0.000	0.030	0.000	0.130	0.000	0.100
Pseudo R^2^	0.005	0.028	0.010	0.032	0.003	0.028

Standard errors are in parentheses; *** *p* < 0.01, ** *p* < 0.05, * *p* < 0.1; LR chi2 demonstrates the joint significance of both independent and control variables, and a *p*-value below 0.1 indicates a high level of joint significance—as shown, all models are jointly significant; Pearson chi2 demonstrates the model’s goodness of fit of the, and when its *p*-value exceeds 0.1, the satisfactory fit of the model is indicated—as shown, the majority of models do not show an ideal goodness of fit, which could be attributed to the insufficient inclusion of influencing factors in the model.

**Table 6:** Regression results simultaneously incorporating factors across multiple levels.

	Individual-Level Factors	Family-Level Factors	Social-Level Factors	All Factors
	(1)	(2)	(3)	(4)
Ability	−0.040 ***			−0.040 ***
	(0.004)			(0.004)
Confidence	−0.158 ***			−0.159 ***
	(0.024)			(0.024)
Attitude	−0.090 ***			−0.078 ***
	(0.011)			(0.012)
Only-child		−0.133 **		−0.136 **
		(0.056)		(0.057)
Consumption		−0.068 ***		−0.057 **
		(0.023)		(0.024)
Relative-NEET		−0.225 ***		−0.199 ***
		(0.056)		(0.057)
Service			−0.302 ***	−0.207 **
			(0.089)	(0.091)
Num-service			−0.100 ***	−0.089 ***
			(0.013)	(0.013)
Loan			−0.417 ***	−0.458 ***
			(0.057)	(0.058)
Gender	0.237 ***	0.212 ***	0.196 ***	0.232 ***
	(0.045)	(0.044)	(0.045)	(0.046)
Age	−0.095 ***	−0.088 ***	−0.090 ***	−0.089 ***
	(0.017)	(0.017)	(0.017)	(0.017)
Nationality	−0.413 ***	−0.365 ***	−0.285 ***	−0.313 ***
	(0.063)	(0.062)	(0.063)	(0.064)
Marriage	−0.570	−0.509	−0.522	−0.461
	(0.368)	(0.359)	(0.361)	(0.367)
Registration	−0.631 ***	−0.641 ***	−0.681 ***	−0.597 ***
	(0.057)	(0.060)	(0.057)	(0.061)
Family-income	−0.066 ***		−0.083 ***	
	(0.016)		(0.016)	
_cons	2.675 ***	0.818 **	1.464 ***	3.081 ***
	(0.350)	(0.330)	(0.341)	(0.360)
N	12,616	12,616	12,616	12,616
Log likelihood	−6333.36	−6471.80	−6391.03	−6261.01
LR chi2	598.99	322.12	483.67	743.69
Prob > chi2	0.000	0.000	0.000	0.000
Pearson chi2	7951.05	1151.71	2189.01	11,715.70
Prob > chi2	0.8212	0.041	0.654	0.630
Pseudo R^2^	0.045	0.024	0.036	0.056

Standard errors are in parentheses; *** *p* < 0.01, ** *p* < 0.05; Family-level is not introduced in Columns (2) and (4) because it is highly correlated with Consumption. Dependence is not included because it is automatically culled when logit regression is used—as shown, the *p*-values of Pearson chi2 for all columns (except Column (2) are found to be significantly greater than 0.1, demonstrating a strong fit between them.

## Data Availability

Where data is unavailable due to privacy or ethical restrictions.
